# Multiple Dimensions of Environmental Justice and Oil and Gas Development in Pennsylvania

**DOI:** 10.1089/env.2022.0041

**Published:** 2024-02-07

**Authors:** Wil Lieberman-Cribbin, Xin Fang, Rachel Morello-Frosch, David J.X. Gonzalez, Elaine Hill, Nicole C. Deziel, Jonathan J. Buonocore, Joan A. Casey

**Affiliations:** Mr. Wil Lieberman-Cribbin is a doctoral student at Department of Environmental Health Sciences, Columbia University Mailman School of Public Health, New York, USA.; Ms. Xin Fang is a Research Assistant at Department of Environmental Health Sciences, Columbia University Mailman School of Public Health, New York, USA.; Dr. Rachel Morello-Frosch is a Professor at Department of Environmental Science, Policy and Management & School of Public Health, University of California, Berkeley, Berkeley, California, USA.; Dr. David J.X. Gonzalez is a postdoctoral fellow at Department of Environmental Science, Policy and Management & School of Public Health, University of California, Berkeley, Berkeley, California, USA.; Dr. Elaine Hill is an Associate Professor at Department of Public Health Sciences, University of Rochester School of Medicine and Dentistry, Rochester, New York, USA.; Dr. Nicole C. Deziel is an Associate Professor at Department of Environmental Health Sciences, Yale University School of Public Health, New Haven, Connecticut, USA.; Dr. Jonathan J. Buonocore is a Research Associate at Center for Climate, Health, and the Global Environment, Harvard University T.H. Chan School of Public Health, Boston, Massachusetts, USA.; Dr. Joan A. Casey is an Assistant Professor at Department of Environmental Health Sciences, Columbia University Mailman School of Public Health, New York, USA.

**Keywords:** environmental justice, social factors, hydraulic fracturing, natural gas, proximity

## Abstract

**Background:**

Community socioeconomic deprivation (CSD) may be related to higher oil and natural gas development (OGD) exposure. We tested for distributive and benefit-sharing environmental injustice in Pennsylvania's Marcellus Shale by examining (1) whether OGD and waste disposal occurred disproportionately in more deprived communities and (2) discordance between the location of land leased for OGD and where oil and gas rights owners resided.

**Materials and Methods:**

Analyses took place at the county subdivision level and considered OGD wells, waste disposal, and land lease agreement locations from 2005 to 2019. Using 2005–2009 American Community Survey data, we created a CSD index relevant to community vulnerability in suburban/rural areas.

**Results:**

In adjusted regression models accounting for spatial dependence, we observed no association between the CSD index and conventional or unconventional drilled well presence. However, a higher CSD index was linearly associated with odds of a subdivision having an OGD waste disposal site and receiving a larger volume of waste. A higher percentage of oil and gas rights owners lived in the same county subdivision as leased land when the community was least versus most deprived (66% vs. 56% in same county subdivision), suggesting that individuals in more deprived communities were less likely to financially benefit from OGD exposure.

**Discussion and Conclusions:**

We observed distributive environmental injustice with respect to well waste disposal and benefit-sharing environmental injustice related to oil and rights owner's residential locations across Pennsylvania's Marcellus Shale. These results add evidence of a disparity between exposure and benefits resulting from OGD.

## INTRODUCTION

Pennsylvania has a long history of drilling for oil and natural gas, with the first oil well drilled in 1859 and the first natural gas production beginning in 1881.^1^ This type of conventional drilling continued to be common in Pennsylvania throughout the 1900s. Since the early 2000s, advancements in horizontal drilling and high-volume hydraulic fracturing enabled natural gas production and extraction from previously inaccessible reservoirs.^2^ This drilling is known as unconventional, characterized by greater drilling depth, directional drilling, specific geological formations, as well as large volumes of high-pressure water, proppants, and chemicals injected into formations to extract natural gas.^2^^,^^3^

In contrast, conventional drilling mainly utilizes vertical drilling as opposed to directional drilling with hydraulic fracturing. The scope of modern unconventional production has led to increased well pad sizes with more wells per pad, wastewater pits, and more processing and transportation equipment than conventional development.^2^^,^^4^^,^^5^ In 2014, more than 1 million Pennsylvanians lived within 1 mile of an active oil or gas well.^6^

Oil and natural gas development (OGD) may harm health through contamination of soil and surface water, noise pollution through drilling operations, and elevated air pollution around well sites.^7^^,^^8^^,^^9^^,^^10^ Research has also noted increased motor vehicle crashes in Pennsylvania counties with drilling.^11^ Proximity and density of OGD have been associated with a range of adverse health outcomes.^9^^,^^12^ These include increased asthma diagnoses,^13^ hospitalizations,^10^^,^^14^^,^^15^^,^^16^ and medication orders,^15,16^ as well as cardiovascular disease.^17^^,^^18^^,^^19^ The largest body of work links increased proximity to OGD with adverse birth outcomes, including preterm birth,^20^^,^^21^^,^^22^^,^^23^ low birth weight,^24^^,^^25^ and congenital heart defects.^17^^,^^26^^,^^27^

Both conventional and unconventional OGD can result in community-level stressors,^28^^,^^29^^,^^30^ such as chronic stress, feelings of powerlessness, tension between residents who support and oppose drilling, and distrust between residents and governing bodies.^30^ Among expecting mothers in Pennsylvania, Casey *et al.* found an association between proximity to more productive unconventional wells and increased risk of depression and anxiety.^31^ Adults living in proximity to more unconventional natural gas development in Pennsylvania also reported increased depression symptoms.^32^

The impacts of OGD have been studied with an environmental justice framework, responding to residents of affected communities who have called attention to perceived environmental racism and classism associated with the siting of oil and gas infrastructure.^2^^,^^33^^,^^34^ Most studies have assessed distributive environmental justice by seeking to determine if racially and socioeconomically marginalized populations face a disproportionate burden of OGD exposure.

In Texas, wastewater wells were more likely to be located in higher poverty areas and in communities of color,^35^ whereas areas with majority versus smaller Hispanic populations were more likely to be exposed to flaring and subsequent air pollution.^22^ Others have looked at procedural environmental justice by assessing whether community stakeholders and populations, including marginalized groups, affected by OGD have a say in decision-making processes.^2,33^ Studies of benefit-sharing injustices have examined the disconnect between who receives economic incentives from OGD and those who are in the path of potentially hazardous emissions.

Several studies have focused on environmental justice related to unconventional drilling in Pennsylvania. In the Marcellus Shale, Ogneva-Himmelberger and Huang used spatial coincidence and proximity metrics and reported that census tracts with a higher proportion of individuals living in poverty were more likely to be exposed to actively producing unconventional natural gas wells from 2002 to 2013.^36^ However, a separate analysis at the block group level using 2013 data did not corroborate this relationship in Pennsylvania.^37^ Clark *et al.* found that higher median household income and educational attainment at the county level were associated with an increased likelihood of submitting an oil and gas complaint in Pennsylvania.^38^ The team also found that the odds of an oil and gas complaint subsequently being investigated and confirmed were lower in counties with higher proportions of minority (Black, Asian, and Native American) populations.^38^

Those arguing for OGD often cite economic benefits, for example, that the oil and gas industry contributed to nearly 10% of Pennsylvania's gross domestic product in 2019.^39^ Furthermore, drilling in the Pennsylvania Marcellus Shale appears to have resulted in modest job growth and increased income, school funding, and wages for non-college-educated men in communities in drilled areas.^37^^,^^40^^,^^41^^,^^42^ However, these benefits may not accrue evenly or equitably among those bearing the brunt of drilling-related exposure. Several studies have examined benefit-sharing injustice in Pennsylvania, centered around mineral rights ownership and lease rights. Inequities can arise from at least two sources.

First, in Pennsylvania, estates can be “split,” meaning the owner of surface and mineral (subsurface location of oil and gas) rights differ. This can lead to a situation where the mineral rights owner receiving royalty payments from OGD does not live on the affected surface land or even own it. In a study of 11 Pennsylvania counties, Kelsey *et al.* found that more than 25% of land was owned by entities residing outside the county where drilling occurred.^37^^,^^43^ Second, mineral rights ownership is often concentrated in a small group of individuals. Focusing on Bradford County, where the greatest number of unconventional wells was drilled in Pennsylvania between 2007 and 2011, Kelsey *et al.* also reported that the top 10% of residential landowners in terms of acreage owned 73% of locally owned land.^43^

This disproportionate mineral rights ownership may have translated into economic inequalities. Between 2007 and 2010, fewer than 10% of residents living in Pennsylvania counties with at least 90 drilled wells reported receiving rents or royalty income.^44^ These findings illustrate the complex pathways through which environmental injustice may operate with respect to OGD and the importance of continued environmental justice analyses as OGD continues in Pennsylvania and elsewhere.

In this article, we examine environmental justice issues from two perspectives. First, to assess distributive justice, we characterize the spatial distribution of OGD in the Pennsylvania Marcellus Shale from both conventional and unconventional drilling between 2005 and 2019. Specifically, we examine the relationship between community socioeconomic deprivation (CSD) and density and proximity of wells relative to population centers, as well as the location of well waste disposal. Second, we use lease agreement data to examine benefit-sharing environmental justice by assessing whether CSD is related to the concordance between the residential location of the oil and gas rights owner and the lease location.

## MATERIALS AND METHODS

We conducted an environmental justice analysis of OGD in Pennsylvania using minor civil divisions (i.e., county subdivisions) as the unit of analysis. We considered county subdivisions “communities” in Pennsylvania as in prior studies.^45^ The study area encompassed the 1848 county subdivisions that overlay the Marcellus Shale in the state. Institutional review board approval was not required as data were publicly available.

### Conventional and unconventional wells

Information on conventional and unconventional wells drilled in the Marcellus Shale from 2005 to 2019 was retrieved from the Carnegie Museum of Natural History's Pennsylvania Unconventional Natural Gas Wells Geodatabase.^46^ This data set provided the American Petroleum Institute (API) well number (identifier), location (latitude/longitude), spud date (initiation of drilling), and classification (conventional or unconventional) of all wells in Pennsylvania. Unconventional wells designated as “gas,” “combined oil and gas,” and “multiple well bore type” were selected as wells for analyses.

We also downloaded data on all wells from Enverus DrillingInfo from 1859 to 2019 to confirm the number of unconventional wells listed in Carnegie data set. The Enverus data set was also used to select the final conventional well data set and identify wells drilled before 2005.^47^ We merged the Carnegie and Enverus (subset to wells drilled 2005–2019) data sets based on API number, filtered out wells designated as unconventional from Carnegie, and considered the remaining wells that produced “oil,” “gas,” or “oil and gas” as conventional. Finally, we created a count of wells drilled before 2005 within each county subdivision using the Enverus data set for use as a potential confounding variable.

### Leasing data

Oil and gas leases are legal contracts between property owners and businesses (e.g., oil and gas companies) and can vary across states. In Pennsylvania, where split-estates are common, the oil and gas rights owner (often referred to as “mineral rights owner”) may not own the surface rights where wells are drilled or live at the site. The oil and gas rights owner signs a lease permitting exploration or drilling by another party. We downloaded leasing data for 2005–2019 from Enverus DrillingInfo.^47^ This data set (*n* = 207,572) provided the latitude and longitude of the county subdivision centroid where land was leased, the oil and gas rights owner and lease recipient names and addresses, and the date and duration of lease agreements. Lease agreements with the Bureau of Land Management and the State of Pennsylvania (*n* = 33), leases pertaining to an area of 0 acres (*n* = 3794), those without an oil and gas rights owner name or address (*n* = 4732), and those with wells missing latitude/longitude coordinates (*n* = 1255) were removed before geocoding.

Lease agreement types were limited to “Leases,” “Lease extensions,” “Memo of Lease,” “Lease option,” and “Lease amendment,” and duplicate records were removed (*n* = 8893). Oil and gas rights owner addresses were geocoded using the “ggmap” package in R and a Google application programming interface key. Of the 188,865 remaining lease records, we were unable to geocode address information for 3230 (1.7%) and excluded these records from analyses. After removing duplicate agreements (mostly due to duplicate date and county subdivision centroids pairs), there were 99,032 parcels of land that were leased during this time period, where a unique parcel was defined as having a unique latitude and longitude centroid and area leased.

### Well waste data

Data on well waste (both liquid and solid), disposal method, and disposal location from 2005 to 2019 were downloaded from the Pennsylvania Geologic Survey's Exploration and Development Well Information Network database.^48^ These data included API number, well type, well latitude and longitude, waste type and quantity, waste reporting period (annual, biannual, or monthly), waste facility name and permit number, and waste facility longitude and latitude. We limited analyses to observations from wells of type “gas,” “combined oil and gas,” and “multiple well bore type.” This original data set contained 1.09 million waste reports from 52,513 wells over the 15-year study period. Of these reports, 128,432 (12%) were missing the waste disposal latitude/longitude. We dropped these observations from analyses. We then excluded observations indicating waste managed via reuse or treatment (*n* = 741,434 [68%]) because these disposal types were less likely to result in community exposure.

We retained disposal types consistent with land application (e.g., “surface impoundment,” “injection disposal well,” “landfill,” “road spreading”). Finally, we considered the location of waste disposal. While Pennsylvania operators export a large portion of waste out of state, the focus of our analyses was Pennsylvania county subdivisions, and thus, we restricted analyses to waste disposed of in-state. Specifically, we identified which subcounties overlaying the Pennsylvania Marcellus Shale received well waste and how much cumulative waste they received. Restricting analyses to waste disposed of within Pennsylvania resulted in a final sample of 111,379 observations from 16,489 wells for analysis. Waste quantity was reported as liquid (barrels [bbls]) and solid (tons) waste; we combined these by estimating waste equivalents in bbls with the conversion of 1 ton waste = 8.5 bbls of waste.

### Urban/rural designations

County subdivisions were classified as “urban” or “rural” according to definitions provided by the Center for Rural Pennsylvania.^49^ This definition is based on population density and relies on 2010 census data to determine the counties that are below (rural) or above (urban) the average population density for the state. All county subdivisions within a given urban/rural county were assigned their respective urban/rural designation. Our study area of the Marcellus Shale contained 404 urban county subdivisions and 1444 rural county subdivisions.

### CSD index

We created an index of CSD at the county subdivision-level using data from the 2005–2009 American Community Survey (ACS),^50^ based on a modified Townsend Index^51^ meant to better measure deprivation in rural communities, by including indicators of low education, poverty, public assistance, and labor force nonparticipation.^45^ Specifically, the CSD index used here includes the proportion of the population with less than a high school education (among those >25 years old), not in the labor force (among those >16 years old), living below the federal poverty threshold (among those >15 years old), unemployed (among civilians >16 years old), households that do not own a car, and households on public assistance.

While the labor force consists of those employed and unemployed, those not in the labor force do not have a job and are not seeking employment and may be an indicator of despair, drug use, or morbidity.^52^^,^^53^ Each variable was z-transformed and summed to create a continuous CSD index measure. Before summing, the proportion of the population in poverty, unemployed, households not owning a car, and households on public assistance was natural log-transformed due to non-normality. Population-weighted centroids were also determined for each county subdivision, relying on data from census block estimates from the year 2000. For analyses, we calculated CSD index quartiles to allow for nonlinearity, where a score of 1 corresponded to the least deprived areas and a score of 4 corresponded to the most deprived (Supplementary Fig. S1).

This quartile classification was performed separately for urban and rural county subdivisions because the CSD index may capture different aspects of deprivation by urban/rural status and because wells were less likely to be drilled in urban areas. As the distribution of unconventional wells was clustered in certain regions across the Marcellus Shale (Fig. 2), we only included counties that contained unconventional wells in the unconventional well analyses to improve comparability of the unexposed communities. Conventional well analyses included all Pennsylvania Marcellus Shale counties.

While we set out to assess the role of residential racial distribution in relation to environmental injustice, the racial composition of areas throughout Pennsylvania is strongly tied to urban and rural designations, and the population of rural Pennsylvania where most wells are located is racially homogeneous and predominantly non-Hispanic White. We therefore opted to focus on socioeconomic status as the metric of community vulnerability and marginalization rather than race/ethnicity, which had limited variability across space.

### Spatial exposure assignment: distributive environmental injustice

We computed a proximity metric and a density metric as surrogates of community-level exposure to conventional and unconventional wells drilled between 2005 and 2019 with which to test for distributive environmental injustice. To estimate the proximity of communities to wells, we identified the distance to the nearest drilled well based on population-weighted centroids (separately for conventional and unconventional wells). To estimate the density of exposure, we drew a 5 km buffer around each county subdivision population-weighted centroid (Supplementary Fig. S2) and counted the number of drilled wells within that buffer. We opted to use population centroids to assign exposure as some rural county subdivisions are quite large with a population clustered in just one portion of the geographic unit. Our method would reduce community-level exposure misclassification.

We estimated exposure to well waste disposed of within Pennsylvania in two ways. First, we created a binary variable scored 1 if a county subdivision ever received well waste and 0 if a county subdivision never received well waste between 2005 and 2019. Second, we summed the total amount of well waste received by each county subdivision in bbl-equivalent between 2005 and 2019. Analyses related to total waste volume were restricted to the *n* = 606 county subdivisions that ever-received waste.

### Spatial exposure assignment: benefit-sharing environmental injustice

**FIG. 1.**
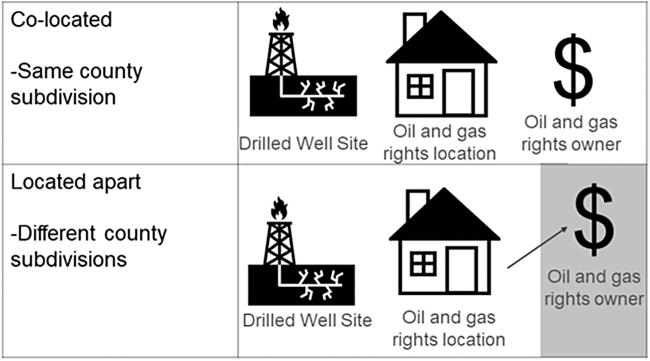
Schematic of the relationship between lease site and oil and gas rights owner residential location. When the lease location and oil and gas rights owners are located apart, the potential arises for benefit-sharing environmental injustice.

For the benefit-sharing environmental justice analysis, we wanted to determine whether there was a relationship between CSD and concordance of oil and gas rights owner's residential location and oil or gas well lease location. We hypothesized that a higher CSD index would correspond to a lower percentage of oil or gas rights owners living in the same county subdivision as the leased land, with therefore less revenue from OGD going to those exposed to drilling. For analyses, we identified the geographic unit (county and county subdivision) of the oil and gas rights owner's address as well as the leased land location (Fig. 1). We used this information to determine if the oil and gas rights owner lived in the same county subdivision as the land they leased (yes/no) and if this relationship differed by CSD index quartile in the county subdivision where the land was leased.

### Statistical analyses

For our first objective assessing distributive justice, we assessed whether the CSD index was associated with community-level exposure to OGD. To evaluate the association between the CSD index with three measures of exposure we used: linear regression (distance to nearest drilled well from population-weighted county subdivision centroids and cumulative volume of well waste deposited in county subdivisions), quasi-Poisson regression (density of drilled wells within a 5 km buffer of county subdivision centroids), and logistic regression (receipt of any well waste vs. no well waste between 2005 and 2019 in each county subdivision).

Each model controlled for county subdivision population density and the presence of any drilled wells before 2005. We ran models separately for exposure to conventional and unconventional wells and by urban and rural classification. Models examining exposure to conventional wells only, unconventional wells only, or unconventional and conventional wells combined were restricted to all county subdivisions belonging to counties where wells (unconventional, conventional, or unconventional and conventional combined, respectively) were drilled. Spatial autocorrelation was accounted for with a tensor spline product of latitude and longitude of county subdivision centroids. We checked models for the presence of residual spatial autocorrelation using semivariograms.^54^

In our second objective evaluating benefit-sharing environmental justice, we created an oil and gas rights ownership-leased land location concordance variable scored 1 if the oil and gas rights owner lived in the same county subdivision as the location of land leased for OGD and scored 0 if the rights owner lived in a different county subdivision. Less concordance indicates worse benefit-sharing environmental justice where those receiving the benefits (rights owners) do not live near the exposure (leased land location). We then determined if the CSD index was related to the likelihood of residential concordance of owner of oil and gas rights location and lease location by comparing percent concordance by CSD index quartile using *χ*^2^-squared tests. All analyses were performed in R Studio version 4.0.2.^55^

## RESULTS

### Well and in-state waste disposal distribution

**FIG. 2.**
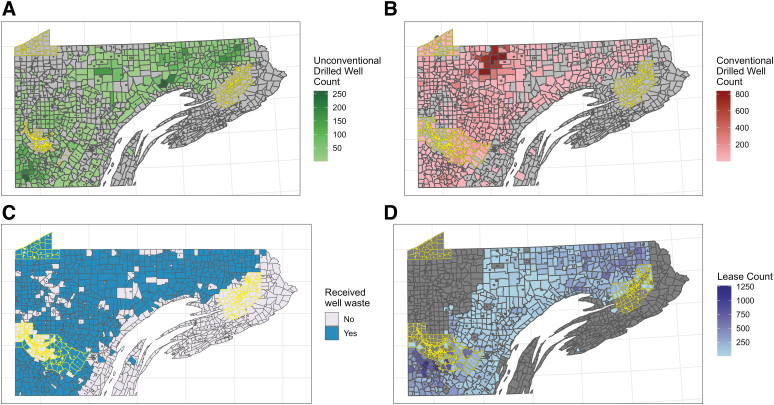
Cumulative spatial distribution from 2005 to 2019 of unconventional wells drilled **(A)**, conventional wells drilled **(B)**, combined unconventional and conventional well waste disposal **(C)**, and oil and gas lease counts **(D)**, according to county subdivisions in the Marcellus Shale. The geographic unit of analysis was the county subdivision and the study area includes those subdivisions that overlay the Marcellus Shale. Unconventional well data (2005–2019) were obtained from the Carnegie Museum of Natural History's Pennsylvania Unconventional Natural Gas Wells Geodatabase and cross-referenced with data from Enverus DrillingInfo. Conventional well data (2005–2019) came from the Enverus DrillingInfo data set. The well waste data came from the Pennsylvania Geologic Survey's Exploration and Development Well Information Network database. Urban county subdivisions are outlined in yellow. County subdivisions without data appear in gray.

Between 2005 and 2019, there were 12,291 unconventional wells drilled and 24,633 conventional wells drilled in county subdivisions (*n* = 1848) that overlap the Marcellus Shale in Pennsylvania. The highest number of conventional wells was drilled in 2006 (*n* = 4411) and 2007 (*n* = 4597), whereas drilling of unconventional wells peaked in 2010 (*n* = 1600) and 2011 (*n* = 1958). These wells were predominantly located in northeast and southwest Pennsylvania (Fig. 2A). While conventional drilling also occurred in these areas (Fig. 2B), conventional wells were drilled more broadly across the study region.

The majority of county subdivisions received waste with larger cumulative volumes of disposal in northeast and southwest Pennsylvania (Fig. 2C). The presence of wells was most common in rural county subdivisions, although some urban areas contained both conventional and unconventional wells (Table 1). Between 2005 and 2019, we identified 7.0 million tons of solid waste and 19 million bbls of liquid waste (equivalent to 78 million bbl total) that were disposed of in Pennsylvania from wells operating in Pennsylvania. In general, waste volume disposed of in Pennsylvania increased over time, with the highest volumes disposed of from 2017 to 2019. The most common in-state land application disposal methods were surface impoundments, road spreading, and landfills.

**Table 1.** Description of Oil and Gas Development Exposure, Community Socioeconomic Deprivation, and Population Density by Urban/Rural Designations and Drilled Well Status of County Subdivisions That Overlay the Pennsylvania Marcellus Shale

	Urban county subdivisions* n* = 404		Rural county subdivisions* n* = 1444	
	Drilled wells	No drilled wells	Drilled wells	No drilled wells
Cumulative unconventional drilled well count 2005–2019, median (IQR)^a^	5.5 (14.0)	0.00 (0.00)	11.0 (33.5)	0.00 (0.00)
Cumulative conventional drilled well count 2005–2019, median (IQR)^b^	6.0 (26.5)	0.00 (0.00)	10.0 (44.2)	0.00 (0.00)
Cumulative waste disposal volume 2005–2019, barrel-equivalent, median (IQR)^c^	11,378 (47,730)	1570 (16,312)	22,246 (125,594)	300 (1091)
CSD index^d^, median (IQR)	−0.26 (2.9)	0.11 (4.6)	1.00 (3.9)	0.92 (4.6)
Population density^e^ (people/km^2^), median (IQR)	115 (297)	594 (1079)	18.1 (29.4)	102 (542)
Spatial area (km^2^), median (IQR)^f^	55.6 (68.8)	4.4 (22.4)	74.7 (54.3)	10.2 (57.3)

^a^ Encompassed 42 urban and 415 rural county subdivisions with drilled unconventional wells, and 362 urban and 1029 rural county subdivisions without unconventional drilled wells.

^b^ Encompassed 86 urban and 504 rural county subdivisions with drilled conventional wells, and 318 urban and 940 rural county subdivisions without conventional drilled wells.

^c^ Encompassed 50 urban and 512 rural county subdivisions with drilled unconventional or conventional wells, and 13 urban and 31 rural county subdivisions without unconventional or conventional drilled wells.

^d^ The index of community socioeconomic deprivation (CSD index) was created at the county subdivision level using data from 2005 to 2009 ACS. Encompassed 101 urban and 627 rural county subdivisions with drilled unconventional or conventional wells, and 303 urban and 817 rural county subdivisions without unconventional or conventional drilled wells.

^e^ Population density was based on total population estimates in county subdivisions from the 2005–2009 ACS. Encompassed 101 urban and 627 rural county subdivisions with drilled unconventional or conventional wells, and 303 urban and 817 rural county subdivisions without unconventional or conventional drilled wells.

^f^ Encompassed 101 urban and 627 rural county subdivisions with drilled unconventional or conventional wells, and 303 urban and 817 rural county subdivisions without unconventional or conventional drilled wells.

ACS, American Community Survey; CSD, community socioeconomic deprivation.

### CSD index

A higher CSD index indicated greater deprivation. With 2005–2009 ACS data, we calculated a median CSD index of 0.69 across the 1848 county subdivisions in the study region. The CSD index showed no consistent spatial pattern (Fig. 2D); however, the CSD index was higher in rural communities (median = 0.96) compared with urban (median = 0.003) communities (Table 1).

### CSD index and exposure to drilled wells and in-state well waste disposal

**FIG. 3.**
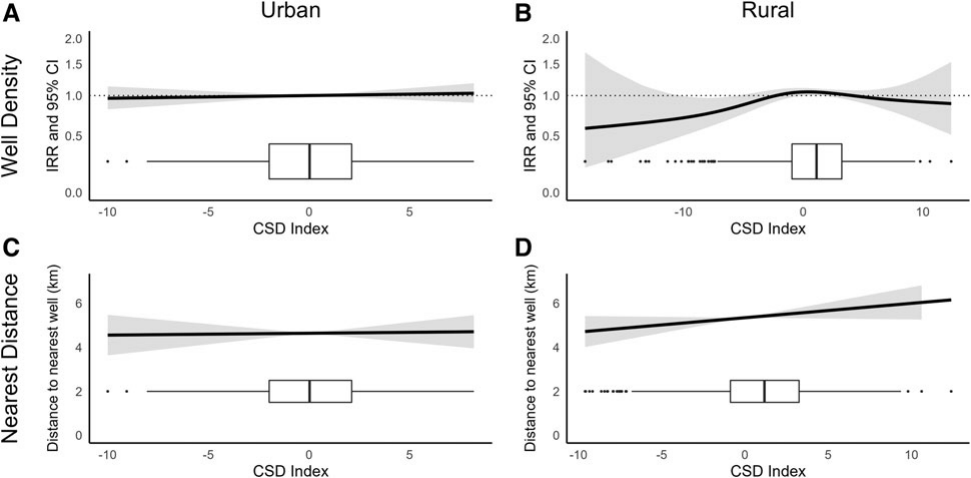
Association between the CSD index and conventional well density in **(A)** urban and **(B)** rural communities and between the CSD index and distance to nearest conventional well in **(C)** urban and **(D)** rural communities among county subdivisions overlaying the Marcellus Shale. Density was modeled with quasi-Poisson regression and proximity with linear regression. Models were adjusted for population density, any wells drilled in the county subdivision before 2005 (yes/no), and a 2D penalized spline for community centroid latitude and longitude. The box plots displayed along the *x*-axis show the distribution of observations across levels of the CSD index. 2D, two-dimensional; CSD, community socioeconomic deprivation.

In analyses utilizing the conventional subset of drilled wells, we observed linear and nonsignificant associations between the CSD index and 5 km conventional well density in urban settings (incidence rate ratio [IRR]: 1.00, 95% confidence interval [CI]: 0.99–1.02) (Fig. 3A). In rural county subdivisions, we observed nonlinear relationships between the CSD index and 5 km conventional well density (CSD index spline *p*-value = 0.16). In rural settings, a higher CSD index was generally associated with higher conventional well density, but CSD index values from −3.0 to 12.4 were associated with a slightly lower conventional well density (Fig. 3B).

**FIG. 4.**
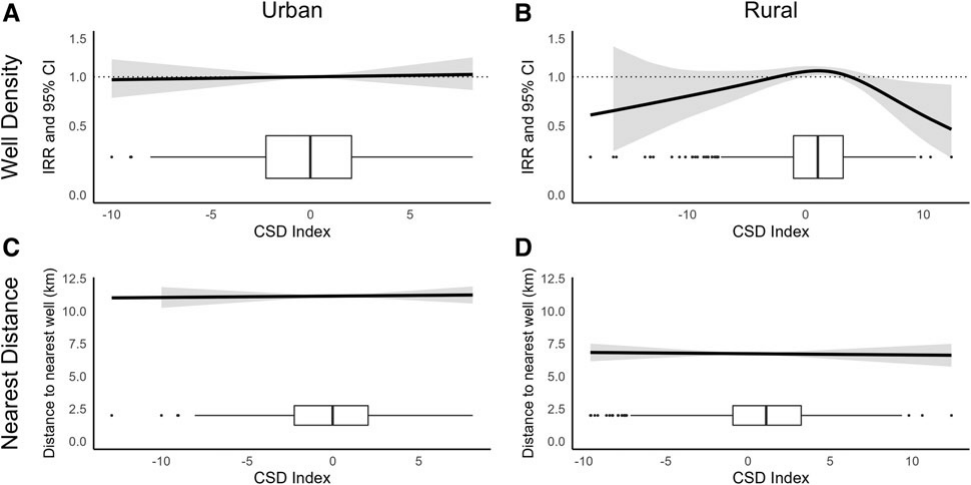
Association between the CSD index and unconventional well density in **(A)** urban and **(B)** rural communities and between the CSD index and distance to nearest unconventional well in **(C)** urban and **(D)** rural communities among county subdivisions overlaying the Marcellus Shale. Density was modeled with quasi-Poisson regression and proximity with linear regression. Models were adjusted for population density, any wells drilled in the county subdivision before 2005, and a 2D penalized spline for community centroid latitude and longitude. The box plots displayed along the *x*-axis show the distribution of observations across levels of the CSD index.

We observed no association between CSD index and nearest well distance in either urban (*β*_adj_: 0.01, 95% CI: −0.08 to 0.10) or rural (*β*_adj_: 0.07, 95% CI: −0.01 to 0.14) settings (Fig. 3C, D). Similarly, we observed no association between the CSD index and 5 km unconventional well density in urban settings (IRR: 1.00, 95% CI: 0.98–1.03; Fig. 4A). We observed a nonlinear association between the CSD index and 5 km unconventional well density in rural settings (*p* = 0.05), and lower unconventional well density at the highest levels of deprivation in rural settings (Fig. 4B).

In nearest distance models for unconventional wells, we did not observe an association in either urban (*β*_adj_: 0.01, 95% CI: −0.07 to 0.09) or rural (*β*_adj_: −0.01, 95% CI: −0.08 to 0.06) settings. In nearest distance models for conventional and unconventional wells combined (Supplementary Fig. S3), no nonlinear associations were observed and the CSD index was again not associated with distance to the nearest drilled well in either urban (*β*_adj_: −0.003, 95% CI: −0.012 to 0.007) or rural (*β*_adj_: 0.001, 95% CI: −0.009 to 0.011) settings. A nonlinear and nonsignificant association was observed between the CSD index and 5 km combined well density in rural settings (*p* = 0.09), with a reduction of well density observed at the highest levels of deprivation (Supplementary Fig. S3). In urban settings, the CSD index was not associated with 5 km well density (IRR: 1.00, 95% CI: 0.99–1.01).

**FIG. 5.**
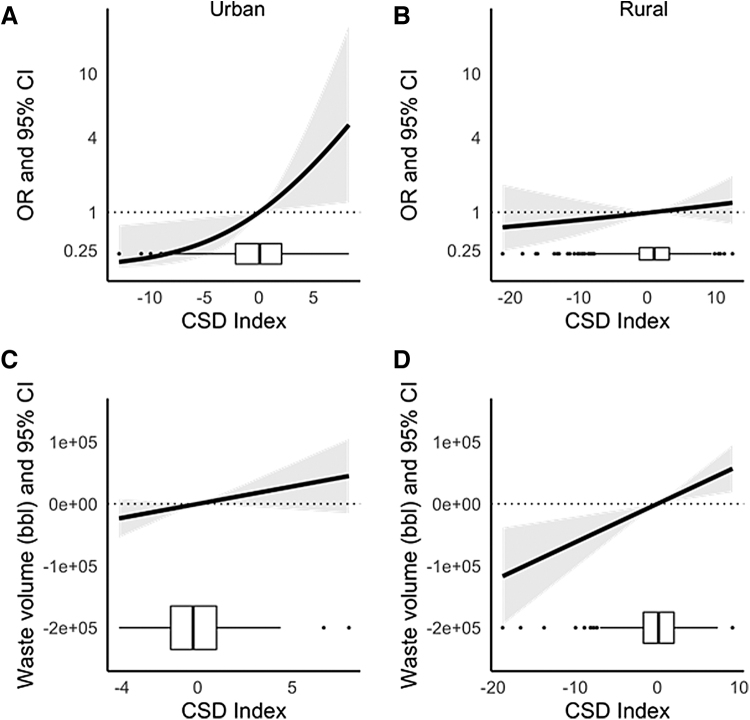
Association between the CSD index and receipt of well waste for disposal (yes/no) in **(A)** urban and **(B)** rural communities and between the CSD index and cumulative volume of well waste for disposal from 2005 to 2019 in **(C)** urban and **(D)** rural communities among county subdivisions overlaying the Marcellus Shale. Models were adjusted for county subdivision population density, any wells drilled in the county subdivision before 2005, and a 2D penalized spline for community centroid latitude and longitude. The box plots displayed along the *x*-axis show the distribution of observations across levels of the CSD index.

In a second set of analyses, we evaluated the relationship between the CSD index and well waste disposal in Pennsylvania county subdivisions. We found that a higher CSD index was linearly associated with higher odds of urban county subdivisions receiving any well waste between 2005 and 2019 (odds ratio [OR] = 1.13, 95% CI: 1.00–1.28) (Fig. 5A). Odds of receiving any waste also increased with higher CSD in rural county subdivisions (OR = 1.02, 95% CI: 0.97–1.08) (Fig. 5B). In analyses that assessed the relationship between the CSD index and cumulative volume of well waste, we restricted to those subdivisions that ever received >0 bbl equivalent of well waste (*n* = 607). We observed linear relationships between the CSD index and the cumulative volume of waste disposed of in urban (β = 5627 bbl-equivalent, 95% CI: −1957 to 13211; Fig. 5C) and rural (β = 6198 bbl-equivalent, 95% CI: 2007–10,390; Fig. 5D) county subdivisions.

### CSD index and concordance between land lease locations and oil and gas rights owner locations

**FIG. 6.**
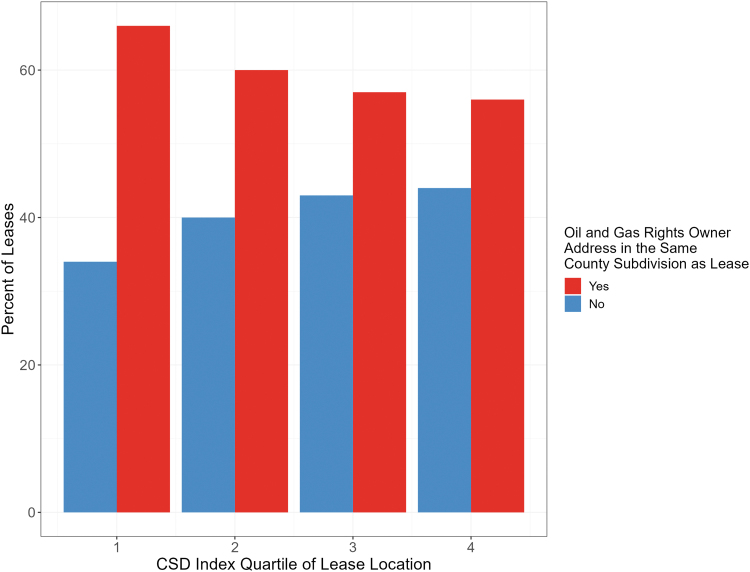
The CSD index quartile and percent of leases in which the oil and gas rights owner address is within the same county subdivision as the lease location. CSD index quartile 1: −21.13 to −1.39; quartile 2: **−**1.40 to 0.68; quartile 3: 0.69 to 2.98; quartile 4: 2.99 to 12.38.

Of the 99,032 lease parcels included in this analysis, 59,279 (59.9%) had an oil and gas rights owner address and a lease location in the same subdivision. We observed a trend where higher deprivation corresponded to a lower proportion of co-located oil and gas rights owners' addresses and leases within county subdivisions (*χ*^2^-squared *p* < 0.01; Fig. 6). In the least deprived areas (CSD index quartile = 1), 66% (*n* = 14,881) of lease parcels had oil and gas rights owners who lived in the same county subdivision, compared with 56% (*n* = 11,388) in the most deprived areas (CSD index quartile = 4) (Fig. 6).

## DISCUSSION

The findings of this multifaceted analysis of OGD in Pennsylvania communities overlying the Marcellus Shale from 2005 to 2019 found that higher levels of the CSD index were associated with increased odds of the community receiving any well waste and of receiving a higher cumulative volume of waste. Furthermore, a higher CSD index in the community where land was leased for drilling was associated with lower likelihood that the entity leasing the land (i.e., benefitting) lived in the OGD-affected community. This latter finding suggests that individuals in more deprived communities were less likely to benefit financially from OGD exposure. We did not observe disparities in exposure to drilled OGD wells, as measured by the CSD index.

This analysis builds on the previous literature by investigating distributive and benefit-sharing environmental justice questions centered on oil and gas drilling in Pennsylvania. Ogneva-Himmelberger *et al.* previously reported in unadjusted analyses that census tracts with a higher proportion of individuals living in poverty had higher exposure to unconventional wells.^36^ We did not find a complementary result using an index of deprivation with adjusted models that accounted for spatial autocorrelation and allowed for nonlinearity. Our results more closely align with Clough and Bell, who did not find evidence that the proportion of people living in poverty was higher in areas closer to active unconventional wells in 2013.^37^

We observed a relationship between higher deprivation and increased odds of any well waste disposal and increased cumulative volume of waste disposal at the county subdivision level in Pennsylvania's Marcellus Shale. To the best of our knowledge, no one has assessed this relationship previously in Pennsylvania. Previous environmental justice studies have found disproportionate waste disposal in areas with higher proportions of racially and socioeconomically marginalized people in the Eagle Ford area of Southern Texas.^35^ In Ohio, disposal wells were more likely to be located in lower income census block groups.^56^ A broad literature describes the legacy of hazardous waste disposal in communities of color and among historically marginalized groups.^57^^,^^58^

We evaluated the range of disposal types in Pennsylvania, which included surface impoundments, landfills, road spreading, and injection disposal wells. Presence of facilities with the capacity to receive waste has previously been associated with lower community socioeconomic status,^59^ and our findings are consistent with this distributive environmental injustice. Such additional waste burden could lead to disproportionate exposure to environmental hazards. Our analyses suggest the need to perform air and drinking water sampling in communities near OGD disposal, where actual or perceived contamination of water can lead to reduced use of tap water.^60^

Increased reliance on bottled water or filtration devices could result in an unfair financial burden among those living near OGD disposal sites. In Pennsylvania, Wrenn *et al.* reported that fracking in the state increased annual bottled water expenditures by more than $19 million in 2010.^61^ A shift toward consuming bottled water integrates with research reporting that wells within 1.5 km of homes reduced the home value of groundwater-dependent homes,^62^ although this effect could differ by region and by home.^63^^,^^64^ Recent work also ties shale gas development to reduced drinking water quality and adverse infant outcomes in Pennsylvania.^23^

While the impacts of wastewater disposal from oil and gas development on water quality have been discussed,^65^^,^^66^ less research has described waste disposal patterns in Pennsylvania.^67^^,^^68^ The present study adds to prior research documenting waste disposal patterns from wells located in Pennsylvania with updated waste disposal volumes summarized through 2019. The volumes of liquid and solid waste disposed in Pennsylvania reported here are within the expected range based on what has been reported previously. We identified 19 million bbls (∼3 billion liters) of liquid waste disposed in Pennsylvania among wells operating in Pennsylvania between 2005 and 2019; this value was equal to the total wastewater generated in the Marcellus Shale from hydraulic fracturing wells in 2011 (3.1 billion liters), indicating that most well waste is disposed of at treatment plants, via reuse, or out of state.^69^

Lutz *et al.* estimated that in 2011 ∼ 1.2 billion liters (∼40%) were disposed of at industrial treatment facilities both within and outside Pennsylvania.^67^ Another group estimated that ∼390 million liters of Marcellus wastewater generated in Pennsylvania were disposed to wastewater treatment plants in Pennsylvania in 2011.^65^ Common reported waste disposal methods include “sent to brine or industrial waste treatment plant,” “injection disposal well,” “landfill,” “municipal sewage treatment plant,” “road spreading,” “reuse at a well site,” and “unknown disposal method.”^65^ In the present environmental justice analysis, we included only land application waste types that we believed posed the greatest health risk to populations living nearby, meaning that our waste summaries are not directly comparable to previous analyses that included all disposal types and waste exported from Pennsylvania.

Our result highlighting the relationship between higher deprivation and higher odds of receiving well waste is particularly relevant considering environmental violations due to OGD, especially those related to wastewater^70^ and water supply complaints.^38^ Major waste types from conventional and unconventional wells include drill cuttings (solid waste), flowback wastewater, and produced brine.^67^ From 2008 to 2011, there was a fourfold increase in produced water from conventional and unconventional wells combined, with Marcellus wells accounting for 79% of produced water by 2011.^67^ A separate analysis found that while injection disposal of wastewater tripled from the beginning to the end of 2011, primarily driven by wells in Southwestern Pennsylvania, reuse was the dominant disposal method in Northeastern Pennsylvania.^68^

We also found that most well waste in Pennsylvania was treated or reused, but that more than 16,000 wells from 2005 to 2019 produced waste that was disposed of within Pennsylvania and that the highest volumes were disposed of in 2017–2019. Prior research has emphasized the potential impacts of wastewater disposal on surface and groundwater quality,^65^^,^^71^ and our results underscore the need to study where disposal occurs and how it influences human health. However, data availability and quality issues remain.

We identified numerous instances of incomplete or missing data regarding the location of waste disposal and disposal methods in production and waste reports. For example, 12% of waste reports did not include a waste disposal latitude/longitude, which is a barrier in the study of trends, patterns, and environmental justice issues related to waste disposal. Missing data can hamper research used to inform energy policy and may also indicate issues of procedural injustice, infringing on the public's right to comprehensive, updated, and accurate information. Future research should explore patterns of waste disposal data availability in Pennsylvania.

Our finding of discordance among lease agreements in Pennsylvania, especially in the most deprived areas, call into question if those who are profiting from land leased also live and are potentially exposed to the adverse consequences of OGD. Prior literature on resource extraction speaks to a possible resource curse where places rich in natural resources experience increased poverty or slowed economic development.^72^ Furthermore, when OGD does result in economic benefit, studies suggest that it may not be felt equally and that those living closest to wells often experience the brunt of the exposure while profiting less.^73^ For example, Kelsey *et al.* reported that in 10 heavily developed Marcellus counties, 40% of land was owned by residents of other counties and that nearly 50% of land was owned by the top 10% of county resident landowners.

In Denton, Texas, an exploration of mineral-rights ownership reported that the majority (61.4%) of those who owned mineral rights for drilling and received these financial benefits were not residents of the city.^74^ We identified a potential issue of benefit-sharing environmental injustice measured by discordance among those who own oil and gas rights and those who live near these leased areas. Impacts on home value are another way OGD can unequally affect those living close to wells. A Colorado study reported lower value homes within 500 feet of drilled oil and gas wells, suggesting economic disparities in who bears the greatest impacts of drilling.^75^ In Pennsylvania, shale gas development was found to have a negative impact on property values among groundwater-dependent homes.^63^

However, homes that relied on municipal water experienced small but positive property value impacts from shale gas development, indicating a potential benefit from lease payments for OGD. Other studies have found that substantial economic benefit accrues to a relatively small portion of oil and gas rights holders in Pennsylvania^44^; for each $1 million in oil and gas production at the county level, there was an $80,000 increase in wage income and about one new job created.^76^ Finally, in Pennsylvania (in contrast to Texas and Colorado), oil and gas well production is not taxed as property and thus, for example, do not fund local school districts.^77^ Instead, an impact fee is charged for each unconventional drilled well, which generates revenue for counties, municipalities, and grant programs. In 2012, of $7.3 billion generated in production value from oil and gas, 1.7% of this production value was allocated to local governments as revenue ($202 million from impact fees on unconventional drilled wells). Whether these benefits accrue equitably within counties remains unknown.

This study had limitations. For one, the geographic information of lease data was only available at the county subdivision level, that is, the latitude/longitude of the leased land was not provided. This prevented more refined spatial analyses that could calculate the precise distance between oil and gas rights owners' locations and lease locations and enable investigation of whether an oil and gas rights owners and leaser located in the same subdivision actually resided at the same address. Bias in exposure assignment will be larger in rural areas where county subdivisions are larger in geographic size than in urban areas. We identified discordance in oil and gas rights owner addresses and well lease location by level of community deprivation. In Pennsylvania, this discordance could arise because rights holders choose to live at an alternative location or because of split estates, where those who own the surface rights differ from those owning the oil and gas rights.

We did not have information on surface rights ownership and thus were only able to study discordance in the address of the oil and gas rights owner with well lease location. Exposure to oil and gas wells was based on population centroid data of county subdivisions, as individual-level data were not available. However, this analysis provides an important assessment at the community level, a geographic unit at which policies may be implemented.^78^ The racial/ethnic homogeneity of the population precluded us from exploring environmental justice issues related to race/ethnicity.

Furthermore, all well data, including oil and gas well locations, lease information, and waste disposal required extensive cleaning, and some data were missing or inaccurate. If this missingness was related to CSD, for example, reports were more likely to contain missingness if a well was in a more deprived community, we may have biased our results toward the null. Finally, in general, distributive environmental justice studies have focused on community proximity to well location, as we did herein, rather than proximity to other oil and gas infrastructure, such as pipelines and processing stations.^33^ Future studies may wish to evaluate disparities in exposure to this auxiliary equipment as they can emit health-harming pollutants, such as volatile organic compounds and particulate matter.^7,9^^,^^79^

## CONCLUSIONS

We found evidence of unequitable well waste disposal and an uneven balance between communities exposed to OGD and those receiving the economic benefits in land leased across Pennsylvania Marcellus Shale communities. We did not identify socioeconomic disparities in drilled well locations. These issues of environmental justice have relevance to previous epidemiology studies that have reported worse health outcomes among racially and socioeconomically marginalized populations exposed to OGD in terms of mental health^31,32^ and birth outcomes.^21,22^ Future studies should consider expanding upon these findings to assess other facets of environmental justice as well as investigate mechanisms that have contributed to the observed disparities.

## Supplementary Material

Supplemental data

Supplemental data

Supplemental data

## Notes

^1^
“Pennsylvania Independent Oil & Gas Association.” 2021. <https://pioga.org/education/pa-oil-and-gas/>. (Last accessed on October 8, 2021).

^2^
A. C. Kroepsch, Peter T. Maniloff, John L. Adgate, Lisa M. McKenzie, Katherine L. Dickinson. “Environmental Justice in Unconventional Oil and Natural Gas Drilling and Production: A Critical Review and Research Agenda.” *Environmental Science and Technology* 53 (Jun 2019): 6601–6615.

^3^
Anna J. Willow and Sara Wylie. “Politics, Ecology, and the New Anthropology of Energy: Exploring the Emerging Frontiers of Hydraulic Fracking.” *Journal of Political Ecology* 21 (2014): 222–236.

^4^
“Energy Education—Conventional vs Unconventional Resource. 2019. <https://energyeducation.ca/encyclopedia/Conventional_vs_unconventional_resource>. (Last accessed on October 13, 2021).

^5^
“Act 13 Frequently Asked Questions.” Pennsylvania Department of Environmental Protection. 2012. <https://files.dep.state.pa.us/OilGas/OilGasLandingPageFiles/Act13/Act_13_FAQ.pdf>. (Last accessed on October 13, 2021).

^6^
E. D. Czolowski, Renee L. Santoro, Tanja Srebotnjak, and Seth B. C. Shonkoff. “Toward Consistent Methodology to Quantify Populations in Proximity to Oil and Gas Development: A National Spatial Analysis and Review.” *Environmental Health Perspectives* 125 (Aug 2017): 086004.

^7^
D. O. Carpenter, “Hydraulic Fracturing for Natural Gas: Impact on Health and Environment.” *Reviews on Environmental Health* 31 (Mar 2016): 47–51.

^8^
J. E. Johnston, E. Lim, and H. Roh, “Impact of Upstream Oil Extraction and Environmental Public Health: A Review of the Evidence.” *The Science of the Total Environmental* 657 (Mar 2019): 187–199.

^9^
N. C. Deziel, Eran Brokovich, Itamar Grotto, Cassandra J. Clark, Zohar Barnett-Itzhaki, David Broday, and Keren Agay-Shay. “Unconventional Oil and Gas Development and Health Outcomes: A Scoping Review of the Epidemiological Research.” *Environmental Research* 182 (Mar 2020): 109124.

^10^
M. Willis, Perry Hystad, Alina Denham, and Elaine Hill. “Natural Gas Development, Flaring Practices and Paediatric Asthma Hospitalizations in Texas.” *International Journey of Epidemiology* 49 (Jan 2021): 1883–1896.

^11^
Jove Graham, Jennifer Irving, Xiaoqin Tang, Stephen Sellers, Joshua Crisp, Daniel Horwitz, Lucija Muehlenbachs, Alan Krupnick, and David Carey. “Increased Traffic Accident Rates Associated with Shale Gas Drilling in Pennsylvania.” *Accident: Analysis and Prevention* 74 (Jan 2015): 203–209.

^12^
K. M. Wollin, G. Damm, H. Foth, A. Freyberger, T. Gebel, A. Mangerich, U. Gundert-Remy, F. Partosch, C. Röhl, T. Schupp, and Jan G. Hengstler. “Critical Evaluation of Human Health Risks Due to Hydraulic Fracturing in Natural Gas and Petroleum Production.” *Archives of Toxicology* 94 (Apr 2020): 967–1016.

^13^
B. Shamasunder, Ashley Collier-Oxandale, Jessica Blickley, James Sadd, Marissa Chan, Sandy Navarro, Michael Hannigan, and Nicole J. Wong. “Community-Based Health and Exposure Study around Urban Oil Developments in South Los Angeles.” *International Journal of Environmental Research Public Health* 15 (Jan 2018):138.

^14^
L. Peng, C. Meyerhoefer, and S. Y. Chou. “The Health Implications of Unconventional Natural Gas Development in Pennsylvania.” *Health Economics* 27 (Jun 2018): 956–983.

^15^
S. G. Rasmussen, Elizabeth L. Ogburn, Meredith McCormack, Joan A. Casey, Karen Bandeen-Roche, Dione G. Mercer, Brian S. Schwartz. “Association between Unconventional Natural Gas Development in the Marcellus Shale and Asthma Exacerbations.” *JAMA Internal Medicine* 176 (Sep 2016): 1334–1343.

^16^
K. Koehler, J. Hugh Ellis, Joan A. Casey, David Manthos, Karen Bandeen-Roche, Rutherford Platt, and Brian S. Schwartz. “Exposure Assessment Using Secondary Data Sources in Unconventional Natural Gas Development and Health Studies.” *Environmental Science and Technology* 52 (May 15 2018): 6061–6069.

^17^
L. M. McKenzie, W. Allshouse, and S. Daniels, “Congenital Heart Defects and Intensity of Oil and Gas Well Site Activities in Early Pregnancy.” *Environment International* 132 (Nov 2019): 104949.

^18^
T. P. McAlexander, Karen Bandeen-Roche, Jessie P. Buckley, Jonathan Pollak, Erin D. Michos, John William McEvoy, and Brian S. Schwartz. “Unconventional Natural Gas Development and Hospitalization for Heart failure in Pennsylvania.” *Journal of American College of Cardiology* 76 (Dec 2020): 2862–2874.

^19^
A. Denham, Mary D. Willis, Daniel P. Croft, Linxi Liu, and Elaine L. Hill. “Acute Myocardial Infarction Associated with Unconventional Natural Gas Development: A Natural Experiment. *Environmental Research* 195 (Apr 2021): 110872.

^20^
J. A. Casey, David A. Savitz, Sara G. Rasmussen, Elizabeth L. Ogburn, Jonathan Pollak, Dione G. Mercer, and Brian S. Schwartz. “Unconventional Natural Gas Development and Birth Outcomes in Pennsylvania, USA.” *Epidemiology* 27 (Mar 2016): 163–172.

^21^
D. J. X. Gonzalez, Allison R. Sherris, Wei Yang, David K. Stevenson, Amy M. Padula, Michael Baiocchi, Marshall Burke, Mark R. Cullen, and Gary M. Shaw. “Oil and Gas Production and Spontaneous Preterm Birth in the San Joaquin Valley, CA: A Case-Control Study.” *Environmental Epidemiology* 4 (Aug 2020): e099.

^22^
L. J. Cushing, Kate Vavra-Musser, Khang Chau, Meredith Franklin, Jill E. Johnston. “Flaring from Unconventional Oil and Gas Development and Birth Outcomes in the Eagle Ford Shale in South Texas. *Environmental Health Perspectives* 128 (Jul 2020): 77003.

^23^
Elaine L. Hill and Lala Ma, “Drinking Water, Fracking, and Infant Health.” *Journal of Health Economics* 82 (2022): 102595.

^24^
K. V. Tran, Joan A. Casey, Lara J. Cushing, and Rachel Morello-Frosch.“Residential Proximity to Oil and Gas Development and Birth Outcomes in California: A Retrospective Cohort Study of 2006–2015 Births.” *Environmental Health Perspectives* 128 (Jun 2020): 67001.

^25^
Elaine L. Hill. “Shale Gas Development and Infant Health: Evidence from Pennsylvania.” *Journal of health economics* 61 (2018): 134–150.

^26^
L. M. McKenzie, Ruixin Guo, Roxana Z. Witter, David A. Savitz, Lee S. Newman, and John L. Adgate. “Birth Outcomes and Maternal Residential Proximity to Natural Gas Development in Rural Colorado.” *Environmental Health Perspectives* 122 (Apr 2014): 412–417.

^27^
Ian W. Tang, Peter H. Langlois, and Verónica M. Vieira, “Birth Defects and Unconventional Natural Gas Developments in Texas, 1999–2011.” *Environmental Research* 194 (2021): 110511.

^28^
R. Z. Witter, Lisa McKenzie, Kaylan E. Stinson, Kenneth Scott, Lee S. Newman, and John Adgate. “The Use of Health Impact Assessment for a Community Undergoing Natural Gas Development. *American Journal of Public Health* 103 (Jun 2013): 1002–1010.

^29^
S. A. Malin. “Depressed Democracy, Environmental Injustice: Exploring the Negative Mental Health Implications of Unconventional Oil and Gas Production in the United States. *Energy Research & Social Science* 70 (Dec 2020): 101720.

^30^
M. Soyer, K. Kaminski, and S. Ziyanak. “Socio-Psychological Impacts of Hydraulic Fracturing on Community Health and Well-Being.” *International Journal of Environmental Research and Public Health* 17 (Feb 2020): 1186.

^31^
J. A. Casey, Dana E. Goin, Kara E. Rudolph, Brian S. Schwartz, Dione Mercer, Holly Elser, Ellen A. Eisen, and Rachel Morello-Frosch. “Unconventional Natural Gas Development and Adverse Birth Outcomes in Pennsylvania: The Potential Mediating Role of Antenatal Anxiety and Depression. *Environmental Research* 177 (Oct 2019): 108598.

^32^
J. A. Casey, Holly C. Wilcox, Annemarie G. Hirsch, Jonathan Pollak, Brian S. Schwartz.“Associations of Unconventional Natural Gas Development with Depression Symptoms and Disordered Sleep in Pennsylvania. *Scientific Reports* 8 (Jul 2018): 11375.

^33^
Emily Clough. “Environmental Justice and Fracking: A Review.” *Current Opinion in Environmental Science & Health* 3 (2018): 14–18.

^34^
Josiah Edwards, “Living near Drilling Is Deadly. Why Don't California Lawmakers Care?” *The New York Times*, 16 November 2020.

^35^
J. E. Johnston, E. Werder, and D. Sebastian. “Wastewater Disposal Wells, Fracking, and Environmental Injustice in Southern Texas.” *American Journal of Public Health* 106 (Mar 2016): 550–556.

^36^
Yelena Ogneva-Himmelberger and Liyao Huang. “Spatial Distribution of Unconventional Gas Wells and Human Populations in the Marcellus Shale in the United States: Vulnerability Analysis.” *Applied Geography* 60 (2015): 165–174.

^37^
Emily Clough and Derek Bell. “Just Fracking: A Distributive Environmental Justice Analysis of Unconventional Gas Development in Pennsylvania, USA.” *Environmental Research Letters* 11 (2016): 025001.

^38^
C. J. Clark, Joshua L. Warren, Nina Kadan-Lottick, Xiaomei Ma, Michelle L. Bell, James E. Saiers, and Nicole C. Deziel. “Community Concern and Government Response: Identifying Socio-Economic and Demographic Predictors of Oil and Gas Complaints and Drinking Water Impairments in Pennsylvania.” *Energy Research & Social Science* 76 (Jun 2021): 102070.

^39^
PricewaterhouseCoopers. Prepared for The American Petroleum Institute. Impacts of the Oil and Natural Gas Industry on the Us Economy in 2019. 2021. <https://www.api.org/-/media/files/policy/american-energy/pwc/api-pwc-economic-impact-report.pdf>. (Last accessed on October 3, 2022).

^40^
Douglas Wrenn, Tim Kelsey, and Edward Jaenicke, “Resident Vs. Nonresident Employment Associated with Marcellus Shale Development.” *Agricultural and Resource Economics Review* 44 (2015): 1–19.

^41^
Matthew Gardner Kelly and Kai A. Schafft, “A “Resource Curse” for Education?: Deepening Education Disparities in Pennsylvania’s Shale Gas Boomtowns.” *Society & Natural Resources* 34 (2021): 23–39.

^42^
Katie Jo Black, Andrew J. Boslett, Elaine L. Hill, Lala Ma, and Shawn J. McCoy. “Economic, Environmental, and Health Impacts of the Fracking Boom.” *Annual Review of Resource Economics* 13 (2021/10/05 2021): 311–334.

^43^
Tim Kelsey, Alexander Metcalf, and Rodrigo Salcedo. Pennsylvania State University Center for Economic and Community Development. Marcellus Shale: Land Ownership, Local Voice, and the Distribution of Lease and Royalty Dollars. 2012. <http://www.pamatters.com/wp-content/uploads/2012/08/the-data.pdf>. (Last accessed on October 3, 2022).

^44^
Kirsten Hardy and Timothy W. Kelsey. “Local Income Related to Marcellus Shale Activity in Pennsylvania. *Community Development* 46 (2015): 329–340.

^45^
Ann Y. Liu, Frank C. Curriero, Thomas A. Glass, Walter F. Stewart, and Brian S. Schwartz. “Associations of the Burden of Coal Abandoned Mine Lands with Three Dimensions of Community Context in Pennsylvania.” *ISRN Public Health* 2012 (2012): 251201.

^46^
J. Whitacre and J. B. Slyder. Carnegie Museum of Natural History Pennsylvania Unconventional Natural Gas Wells Geodatabase. Pittsburgh, PA: Carnegie Museum of Natural History, 2019. <https://maps.carnegiemnh.org/index.php/projects/unconventional-wells/>. (Last accessed on July 6, 2021).

^47^
“Enverus Drillinginfo.” 2021. <https://app.drillinginfo.com/production/#/default>. (Last accessed on August 2021).

^48^
Pennsylvania Department of Conservation and Natural Resources. Database of Pennsylvania Oil and Gas Wells (EDWIN). 2021. <https://www.dcnr.pa.gov/Geology/GeologicEconomicResources/OilAndGas/Pages/default.aspx>. (Last accessed on July 6, 2021).

^49^
“The Center for Rural Pennsylvania.” 2021. <https://www.rural.palegislature.us/rural_urban.html>. (Last accessed on October 2021).

^50^
Steven Manson, Jonathan Schroeder, David Van Riper, Tracy Kugler, and Steven Ruggles. *Ipums National Historical Geographic Information System: Version 16.0 [Dataset]*. (University of Minnesota, 2021).

^51^
Peter Townsend, Peter Phillimore, and Alastair Beattie. *Health and Deprivation: Inequality and the North* (London: Croom Helm, 1988).

^52^
Anne Case and Angus Deaton. “Rising Morbidity and Mortality in Midlife among White Non-Hispanic Americans in the 21st Century. *Proceedings of the National Academy of Sciences* 112 (2015): 15078–15083.

^53^
Iliya Gutin and Robert A. Hummer. “Occupation, Employment Status, and “Despair”-Associated Mortality Risk among Working-Aged US Adults, 1997–2015.” *Preventive Medicine* 137 (2020): 106129.

^54^
Noel Cressie. *Statistics for Spatial Data*. (John Wiley & Sons, 2015).

^55^
*R: A Language and Environment for Statistical Computing*. (R Foundation for Statistical Computing, Vienna, Austria, 2013).

^56^
Genevieve S. Silva, Joshua L. Warren, and Nicole C. Deziel, “Spatial Modeling to Identify Sociodemographic Predictors of Hydraulic Fracturing Wastewater Injection Wells in Ohio Census Block Groups.” *Environmental Health Perspectives* 126 (2018): 067008.

^57^
Robert D. Bullard, Paul Mohai, Robin Saha, and Beverly Wright.“Toxic Wastes and Race at Twenty: Why Race Still Matters after All of These Years.” *Environmental Law* 38 (2008): 371.

^58^
Charles Lee. *Toxic Waste and Race in the United States.* (New York, NY: Routledge, 2019).

^59^
Spencer Banzhaf, Lala Ma, and Christopher Timmins. “Environmental Justice: The Economics of Race, Place, and Pollution.” *Journal of Economic Perspectives* 33 (2019): 185–208.

^60^
Cassandra J. Clark, Boya Xiong, Mario A. Soriano Jr, Kristina Gutchess, Helen G. Siegel, Emma C. Ryan, Nicholaus P. Johnson, Kelsie Cassell, Elise G. Elliott, Yunpo Li, Austin J. Cox, Nicolette Bugher, Lukas Glist, Rebecca J. Brenneis, Keli M. Sorrentino, Julie Plano, Xiaomei Ma, Joshua L. Warren, Desiree L. Plata, James E. Saiers, and Nicole C. Deziel.“Assessing Unconventional Oil and Gas Exposure in the Appalachian Basin: Comparison of Exposure Surrogates and Residential Drinking Water Measurements. *Environmental Science & Technology* 56 (2022): 1091–1103.

^61^
Douglas H. Wrenn, H. Allen Klaiber, and Edward C. Jaenicke. “Unconventional Shale Gas Development, Risk Perceptions, and Averting Behavior: Evidence from Bottled Water Purchases. *Journal of the Association of Environmental Resource Economists* 3 (2016): 779–817.

^62^
Lucija Muehlenbachs, Elisheba Spiller, and Christopher Timmins. “The Housing Market Impacts of Shale Gas Development. *American Economic Review* 105 (2015): 3633–3659.

^63^
Sathya Gopalakrishnan and H. Allen Klaiber “Is the Shale Energy Boom a Bust for Nearby Residents? Evidence from Housing Values in Pennsylvania.” *American Journal of Agricultural Economics* 96 (2014): 43–66.

^64^
Andrew Boslett, Todd Guilfoos, and Corey Lang, “Valuation of Expectations: A Hedonic Study of Shale Gas Development and New York's Moratorium.” *Journal of Environmental Economics Management* 77 (2016): 14–30.

^65^
Nathaniel R. Warner, Cidney A. Christie, Robert B. Jackson, and Avner Vengosh. “Impacts of Shale Gas Wastewater Disposal on Water Quality in Western Pennsylvania.” *Environmental Science Technology* 47 (2013): 11849–11857.

^66^
William D. Burgos, Luis Castillo-Meza, Travis L. Tasker, Thomas J. Geeza, Patrick J. Drohan, Xiaofeng Liu, Joshua D. Landis, Jens Blotevogel, Molly McLaughlin, Thomas Borch, Nathaniel R. Warner.“Watershed-Scale Impacts from Surface Water Disposal of Oil and Gas Wastewater in Western Pennsylvania.” 51 (2017): 8851–8860.

^67^
Kelly O. Maloney and David A. Yoxtheimer, “Production and Disposal of Waste Materials from Gas and Oil Extraction from the Marcellus Shale Play in Pennsylvania," *Environmental Practice* 14 (2012): 278–287.

^68^
Brian G. Rahm, Josephine T. Bates, Lara R. Bertoia, Amy E. Galford, David A. Yoxtheimer, and Susan J. Riha.“Wastewater Management and Marcellus Shale Gas Development: Trends, Drivers, and Planning Implications. *Journal of Environmental Management* 120 (2013): 105–113.

^69^
Brian D. Lutz, Aurana N. Lewis, and Martin W. Doyle. “Generation, Transport, and Disposal of Wastewater Associated with Marcellus Shale Gas Development.” *Water Resources Research* 49 (2013): 647–656.

^70^
Noura Abualfaraj, Mira S.Olson, Patrick L.Gurian, Anneclaire De Roos, and Carol Ann Gross-Davis. “Statistical Analysis of Compliance Violations for Natural Gas Wells in Pennsylvania.” *Energy Policy* 97 (2016): 421–428.

^71^
Jessica M. Wilson and Jeanne M. VanBriesen. “Oil and Gas Produced Water Management and Surface Drinking Water Sources in Pennsylvania.” *Environmental Practice* 14 (2012): 288–300.

^72^
Adam Mayer, Shawn K. Olson-Hazboun, and Stephanie Malin, “Fracking Fortunes: Economic Well-Being and Oil and Gas Development Along the Urban-Rural Continuum.” *Journal of Rural Sociology* 83 (2018): 532–567.

^73^
Jeffrey B. Jacquet, Anne N. Junod, Dylan Bugden, Grace Wildermuth, Joshua T. Fergen, Kirk Jalbert, Brian Rahm, Paige Hagley, Kathryn J. Brasier, Kai Schafft, Leland Glenna, Timothy Kelsey, Joshua Fershee, David L. Kay, and Richard C. Stedman, James Ladlee. “A Decade of Marcellus Shale: Impacts to People, Policy, and Culture from 2008 to 2018 in the Greater Mid-Atlantic Region of the United States.” *The Extractive Industries and Society* 5 (2018/11/01/2018): 596–609.

^74^
Matthew Fry, Adam Briggle, and Jordan Kincaid. “Fracking and Environmental (in) Justice in a Texas City. *Ecological Economics* 117 (2015): 97–107.

^75^
L. M. McKenzie, William B. Allshouse, Troy Burke, Benjamin D. Blair, John L. Adgate.“Population Size, Growth, and Environmental Justice near Oil and Gas Wells in Colorado.” *Environmental Science & Technology* 50 (Nov 2016): 11471–11480.

^76^
James Feyrer, Erin T. Mansur, and Bruce Sacerdote. “Geographic Dispersion of Economic Shocks: Evidence from the Fracking Revolution. *American Economic Review* 107 (2017): 1313–1334.

^77^
Richard G. Newell and Daniel Raimi. “Oil and Gas Revenue Allocation to Local Governments in Eight States. (National Bureau of Economic Research, 2015).

^78^
Nicholaus P. Johnson, Joshua L. Warren, Elise G. Elliott, Linda M. Niccolai, Nicole C. Deziel.“A Multiregion Analysis of Shale Drilling Activity and Rates of Sexually Transmitted Infections in the United States. *Sexually transmitted diseases* 47 (2020): 254–260.

^79^
Robert F. Swarthout, Rachel S. Russo, Yong Zhou, Brandon M. Miller, Brittney Mitchell, Emily Horsman, Eric Lipsky, David C. McCabe, Ellen Baum, Barkley C. Sive.“Impact of Marcellus Shale Natural Gas Development in Southwest Pennsylvania on Volatile Organic Compound Emissions and Regional Air Quality.” *Environmental Science & Technology* 49 (2015/03/03 2015): 3175–3184.

