# Supplementary material for: Multiple Dimensions of Environmental Justice and Oil and Gas Development in Pennsylvania
Source: Environ Justice. 2024 Feb 7;17(1):31–44. doi: 10.1089/env.2022.0041 (PMC10880506; doi:10.1089/env.2022.0041)
Supplement: Supplemental data [file Suppl_FigS1.docx]

Supplementary Figure 1: Spatial distribution of community socioeconomic deprivation (CSD) Index quartiles. The CSD Index was created at the county subdivision-level using data from 2005–2009 American Community Survey.

**
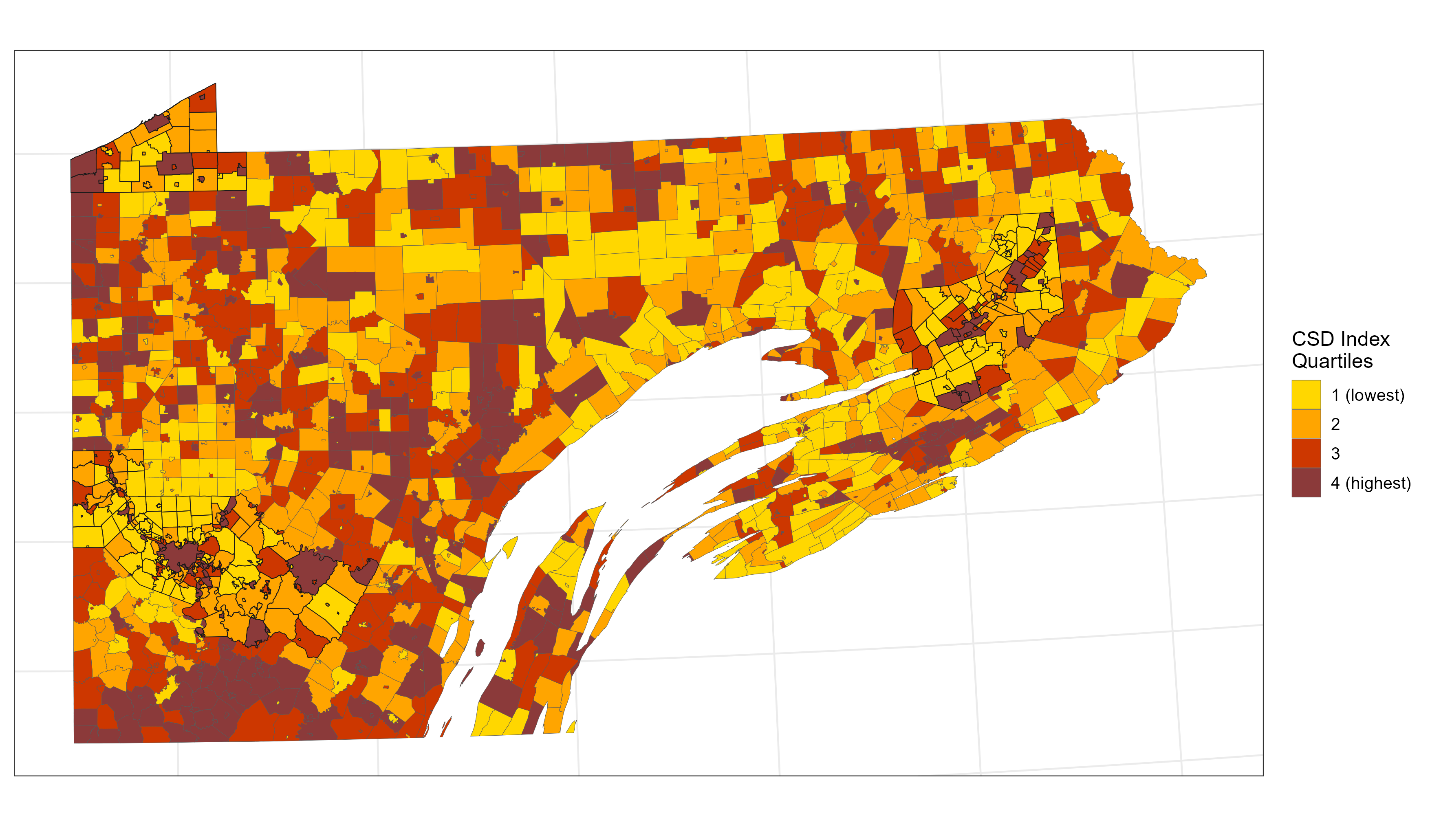
**
