# Supplementary material for: Multiple Dimensions of Environmental Justice and Oil and Gas Development in Pennsylvania
Source: Environ Justice. 2024 Feb 7;17(1):31–44. doi: 10.1089/env.2022.0041 (PMC10880506; doi:10.1089/env.2022.0041)

Supplementary Figure 2: Depiction of 5 km buffers around each county subdivision population-weighted centroid overlaying the Marcellus Shale.


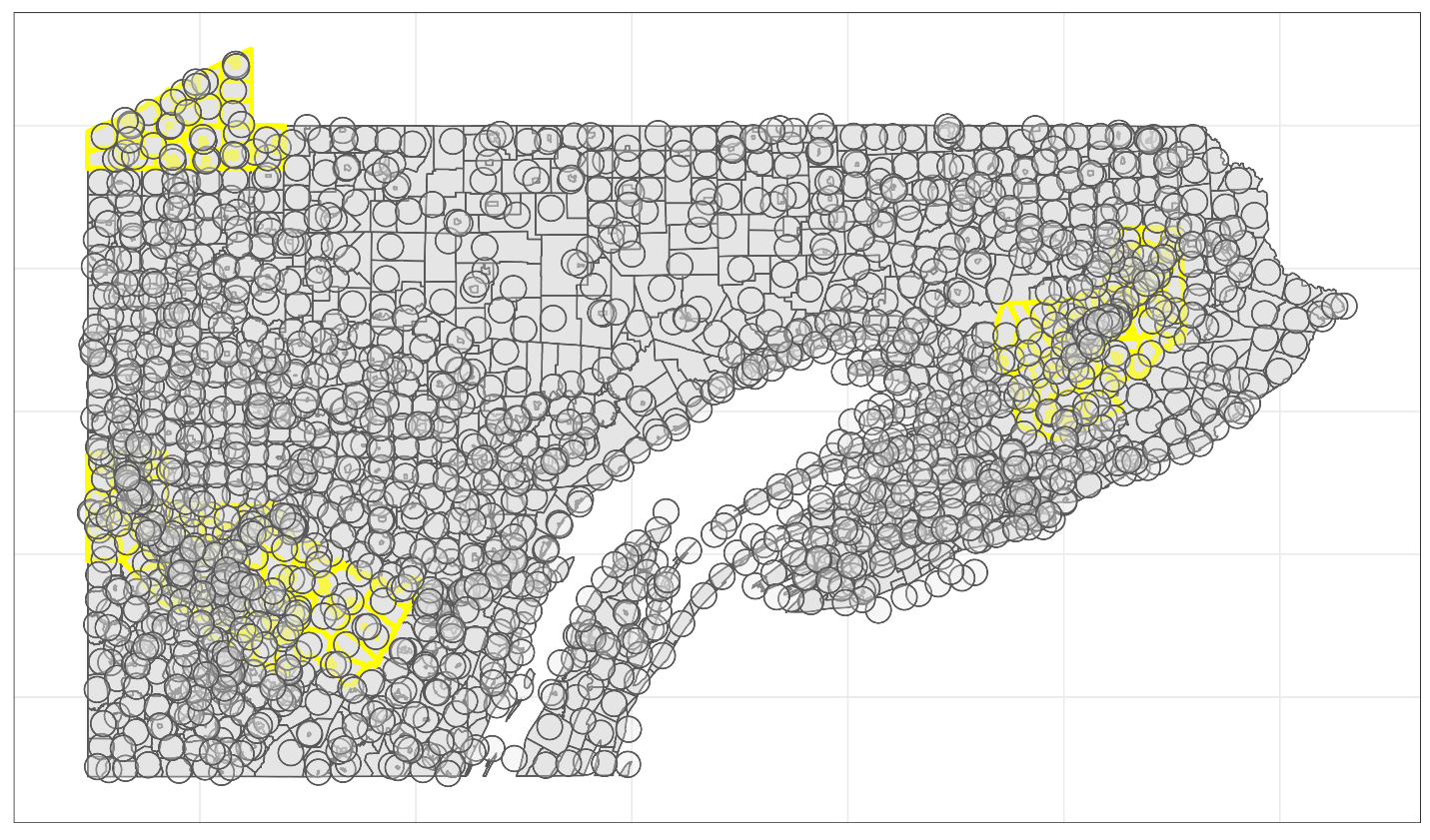

Supplement: Supplemental data [file Suppl_FigS2.docx]
