# Supplementary material for: Multiple Dimensions of Environmental Justice and Oil and Gas Development in Pennsylvania
Source: Environ Justice. 2024 Feb 7;17(1):31–44. doi: 10.1089/env.2022.0041 (PMC10880506; doi:10.1089/env.2022.0041)

Supplementary Figure 3: Association between community socioeconomic deprivation (CSD) and well density (conventional and unconventional wells combined) in A) urban and B) rural communities and between CSD and distance to nearest well in C) urban and D) rural communities in the Marcellus Shale. Density was modeled with quasi-Poisson regression and distance with linear regression. Models were adjusted for population density, prior drilled wells in the county subdivision (yes/no), and a 2-D penalized spline for community latitude and longitude.


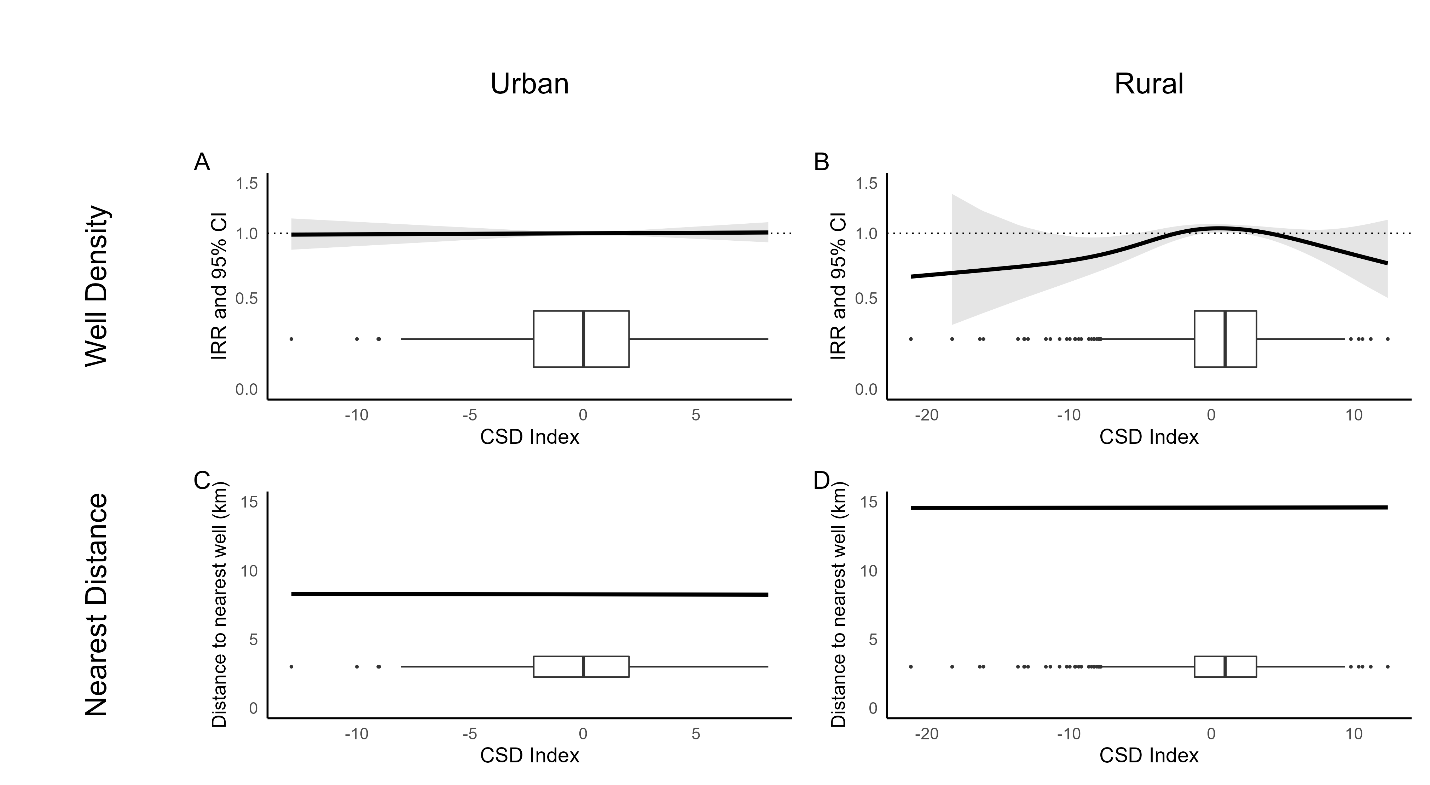

Supplement: Supplemental data [file Suppl_FigS3.docx]
